# Pigmentation phototype and prostate and breast cancer in a select Spanish population—A Mendelian randomization analysis in the MCC-Spain study

**DOI:** 10.1371/journal.pone.0201750

**Published:** 2018-08-14

**Authors:** Inés Gómez-Acebo, Trinidad Dierssen-Sotos, Camilo Palazuelos, Pablo Fernández-Navarro, Gemma Castaño-Vinyals, Jéssica Alonso-Molero, Carmen Urtiaga, Tania Fernández-Villa, Eva Ardanaz, Manuel Rivas-del-Fresno, Ana Molina-Barceló, José-Juan Jiménez-Moleón, Lidia García-Martinez, Pilar Amiano, Paz Rodriguez-Cundin, Víctor Moreno, Beatriz Pérez-Gómez, Nuria Aragonés, Manolis Kogevinas, Marina Pollán, Javier Llorca

**Affiliations:** 1 CIBER Epidemiología y Salud Pública (CIBERESP),Spain; 2 University of Cantabria–IDIVAL, Santander, Spain; 3 Cancer and Environmental Epidemiology Unit, National Center for Epidemiology, Carlos III Institute of Health, Madrid, Spain; 4 Cancer Epidemiology Research Group, Oncology and Hematology Area, IIS Puerta de Hierro (IDIPHIM), Madrid, Spain; 5 ISGlobal Centre for Research in Environmental Epidemiology (CREAL), Barcelona, Spain; 6 Universitat Pompeu Fabra (UPF), Barcelona, Spain; 7 IMIM (Hospital Del Mar Medical Research Institute), Barcelona, Spain; 8 Public Health Division of Gipuzkoa, BioDonostia Research Institute, San Sebastian, Spain; 9 Grupo de Investigación en Interacciones Gen-Ambiente y Salud, Instituto de Biomedicina (IBIOMED), Universidad de León, León, Spain; 10 Navarra Public Health Institute, Pamplona, Spain; 11 IdiSNA, Navarra Institute for Health Research, Pamplona, Spain; 12 Hospital de Cabueñes, Asturias, Spain; 13 Área de Cáncer y Salud Pública, Fundación para el Fomento de la Investigación Sanitaria y Biomédica de la Comunitat Valenciana (FISABIO-Salud Pública), Valencia, Spain; 14 Instituto de Investigación Biosanitaria de Granada (ibs.GRANADA), Granada, Spain; 15 University Hospital Marques de Valdecilla–IDIVAL, Santander, Spain; 16 Cancer Prevention and Control Program, Catalan Institute of Oncology-IDIBELL, L’Hospitalet de Llobregat, Spain; 17 Department of Clinical Sciences, Faculty of Medicine, University of Barcelona, Barcelona, Spain; 18 Subdirección General de Epidemiología, Dirección General de Salud Pública, Consejería de Sanidad, Comunidad de Madrid, Spain; 19 School of Public Health, Athens, Greece; Ohio State University Wexner Medical Center, UNITED STATES

## Abstract

**Introduction:**

Phototype has been associated with an increased risk of prostate cancer, and it is yet unknown if it is related to other hormone-dependent cancers, such as breast cancer or whether this association could be considered causal.

**Methods:**

We examined the association between the phototype and breast and prostate cancers using a Mendelian randomization analysis. We studied 1,738 incident cases of breast cancer and another 817 cases of prostate cancer. To perform a Mendelian randomization analysis on the phototype—cancer relationship, a genetic pigmentation score was required that met the following criteria: (1) the genetic pigmentation score was associated with phototype in controls; (2) the genetic pigmentation score was not associated with confounders in the relationship between phototype and cancer, and (3) the genetic pigmentation score was associated with cancer only through its association with phototype. Once this genetic score is available, the association between genetic pigmentation score and cancer can be identified as the association between phototype and cancer.

**Results:**

The association between the genetic pigmentation score and phototype in controls showed that a higher genetic pigmentation score was associated with fair skin, blond hair, blue eyes and the presence of freckles. Applying the Mendelian randomization analysis, we verified that there was no association between the genetic pigmentation score and cancers of the breast and prostate.

**Conclusions:**

Phototype is not associated with breast or prostate cancer.

## Introduction

Breast and prostate cancer (hormone dependent cancers) are leading causes of cancer related deaths in the Western world. Breast cancer (BC) is the most frequent type of cancer in women worldwide and a main cause of cancer related deaths in developed countries[1] while prostate cancer (PCa) is the second most frequent cancer among men worldwide, following lung cancer. In addition, the incidence of both cancers is growing in both Western [1] and Eastern countries [2] due, on the one hand, to increased exposure to environmental risk factors and the aging of the population and, on the other hand, to the improved diagnostic techniques and generalization of screening.

Epidemiological research has led to the identification of several BC risk factors associated with the production of estrogen (age at menarche, parity, age at first full-term pregnancy, age at menopause),[3,4] and a number of risk factors related to lifestyle (tobacco smoking, alcohol consumption, being overweight or obesity)[5], although their importance seems to be lower than the estrogen-related factors. Risk factors can only explain 40% of the risk of BC. Compared to other common cancers, the etiology of PCa remains a mystery. Although inflammation, diet, physical inactivity, weight, waist circumference and high body mass index (BMI), play a role as risk factors for PCa[6–12], their link to its etiology remain uncertain and the only well-established risk factors for PCa are family history, ethnicity and age[13–17]. Some genetic variants have also been found to be associated with BC and PCa; for instance, variants in BRCA1 and BRCA2 are strongly associated with BC[18,19], and some genetic scores have been developed for both types of cancer[20,21].

### Pigmentation phenotypes

The growth and survival of BC and PCa is regulated by the gonadal steroid hormones. Two therapeutic subtypes of estrogen, ERα and ERβ receptors that mediate the action of estrogen and are factors of nuclear transcription, are considered therapeutic targets for BC and PCa[22]. Estrogens can increase tyrosinase activity and melanin content in normal skin [23]; it is noteworthy that ERb is more frequent in both skin and prostate tissue than ERa[23]. Moreover, estrogens can regulate skin pigmentation via G-protein coupled receptors[24].

As far as we know, there is one study that evaluates the relationship between exposure of pigmentation phenotypes (eye color, hair color, skin phototype, and the presence of freckles, and the way in which exposure to the phenotype was measured) and the incidence of BC[25]. We have also identified two studies that directly examined the relationship between pigmentation phenotypes and the incidence of PCa, another hormone-dependent tumor. On the one hand, Bonilla et al found that British white men who were more likely to have freckles, a lighter skin color and were more prone to burning than tanning had a higher risk of developing PCa than men without such variants[26] However, it is unclear whether these links are coincidental. In fact, the findings may be due to confounders simultaneously associated with the fair skin phototype and cancer, such as ultraviolet radiation (UV) exposure, seasons of the year, level of vitamin D or use of sun cream with high protection. On the other hand, Weinstein et al. paper found no association between eye color and skin phototype[27], but red-haired participants in the study were significantly less likely to develop PCa than men with light brown hair[27].

Moreover, to our knowledge, there are only two prospective studies that investigate the relationship between phenotype and cancer[25,27]; reverse causality, therefore, may explain some associations between cancers and sun exposure, as patients could change their habits after receiving a cancer diagnosis. In this same way, prospective studies do not support the hypothesis that the relationship between personal UV radiations (UVR) lowers the incidence of BC [25]. An information bias could also have occurred, since most of the studies that observed associations between phototype and cancer have used self-reported (subjective) data on hair, skin and eye color and amount of sun exposure sun instead of objectively measured data.

### Mendelian randomization

To solve this problem, we have used the Mendelian randomization analysis [28,29], this method was also used by Bonilla in 2013 [26].This is an epidemiological approach that aims to avoid confounding, measurement problems and reverse causality through the use of genetic variants with effect on a modifiable exposure such as the phenotype of skin, eyes, hair and freckles[30–33]. Mendelian randomization is not influenced by reverse causality because these genetic variants are established at conception and could be considered randomly assigned at conception in relation to probable confounding factors. If the phenotype is truly a causal risk factor in cancer development, it would be expected that genetic variants that modify the phenotype will also increase the risk of cancer. Thus, to assess the relationship between pigmentation traits (eye color, hair color, skin phototype, and the presence of freckles) and the risk of BC and PCa, in this article, we built a genetic score associated with pigmentation characteristics and analyzed its relationship with BC and PCa in a case-control study conducted in Spain.

## Methods

### Study design and population

It is a population-based case-control study carried out in 12 Spanish provinces. This study called Multicase-control (MCC-Spain) evaluates five common types of cancer in Spain (breast, prostate, colorectal, stomach, and chronic lymphocytic leukemia) and has previously been detailed in another article [34].

Cases and controls were recruited between September 1st, 2008 and December 31st, 2013. In this analysis, we included 1,738 incident cases of BC in women and their 1,910 controls, and 817 incident cases of PCa and their 1,006 controls. Subjects were drafted from 23 hospitals and primary care centers across 12 Spanish provinces. All cases had received pathology-confirmed diagnosis; they were between 20 and 85 years old and lived in the vicinity of each hospital at least 6 months before diagnosis. Controls, people from the same catchment area as the cases, without BC or PCa history, were chosen at random from the lists of General Practitioners at primary health centers; they were frequency matched by recruitment province and age (±5 years). Response rates were 72% among cases and 52% among controls. Ethnicity can be a potential variable of confusion, since skin pigmentation is related to ethnicity (perhaps as a consequence of melanosome size and the substantial variation in melanin composition[35]), and ethnicity is related to PCa incidence as well as other variables, all cases and controls selected were white or caucasian.

This study was approved by each of the ethics committees of the participating institutions (the Clinical Research Ethics Committee of Asturias, Barcelona, Cantabria, Girona, Granada, Gipuzkoa, Huelva, León, Madrid, Murcia, Navarra and Valencia). In addition, all the participants included in the study were fully informed and signed a consent form and all procedures performed on humans were carried out in accordance with the ethical standards of the institutional and / or national research committee and taking into account the Declaration of Helsinki of 1964 and its subsequent modifications or comparable ethical standards.

### Data collection

Trained, experienced personnel conducted comprehensive interviews to evaluate sociodemographic information, anthropometric data, smoking habits, alcohol consumption, reproductive history, and personal and family history of cancer of the participants. BMI was calculated taking into account self-reported weight and height records noted one year before diagnosis for cases and one year before the interview for controls. Pigmentation (hair, skin and eye color or freckles) and phototype (classified applying the Fitzpatrick scheme[36], were also self-reported. Behavior of the skin in the sun (classified applying the Fitzpatrick scheme) was categorized into four type of skin (skin type 1: I get tanned easily, I don't get burn; skin type 2: I rarely get burned and then I get tanned; skin type 3: I get burned and then I get tanned and skin type 4: I get burned and almost never I get tanned). Hair color type was categorized into five graded types of category (black or very dark brown, brown, light brown and blonde). Eye color was divided into three categories, blue or grey, green or light brown and dark brown.

### Genotyping and SNP selection for the genetic risk scores

Blood samples were obtained following the study protocol[37]. The genotyping was carried out using the Infinium Human Exome BeadChip (Illumina, San Diego, USA) which is made up of >200,000coding markers plus 5,000 additional custom SNPs chosen from prior genome-wide association studies (GWAS) or in genes of interest. The principal components were estimated using the PLINK—cluster command, which implements complete linkage agglomerative clustering based on pairwise identity-by-state distance.

Genetic variants linked to pigmentation phenotypes in GWAS were identified using GWAS catalog Genomebrowser [38] after searching for pigmentation phenotypes as “reported trait” with a p-value threshold of 5×10^−8^, evident in European ancestry (initial or replication sample). For this analysis, 18 of these pigmentation susceptibility variants could be included. Finally, rs12931267 had to be eliminated given that it was in linkage disequilibrium with rs1805007 SNPs (r^2^ >0.8). In accordance with Hardy-Weinberg equilibrium (HWE) no SNP was rejected as no SNP achieved statistical significance (p < 10^−4^) in the HWE test. The complete list of 17 SNPs obtained from the literature is detailed in S1 Table. In order to build a genetic pigmentation score (GPS), genotypes of these 17 SNPs were codified as 0, 1 or 2 and added for obtaining the GPS in such a way that higher scores were associated with light-color eyes, blonde hair, fairer skin, freckles, and skin getting more easily burned or tanned; finally, the SNP codes. Note that, although our GPS is constituted by the SNPs selected from the literature, the risk allele for each variant originates from a SNP by SNP analysis with our data, hence it may not match that reported originally. Such a SNP by SNP analysis tests for the association between each variant and the 5 phototype traits discussed earlier, even if no association between the SNP and a particular trait has been published. For example, rs1015362 is associated with hair colour, freckles and tanning in the literature, whereas in our data it shows a significant association with eye colour.

### Statistical analysis

For performing a Mendelian randomization analysis on phototype–cancer relationship, the GPS needs to meet with the following criteria: (1) the GPS is associated with phototype in controls; (2) the GPS is independent of confounders; and (3) the GPS is not associated with cancer conditional on the phototype and confounders of the relationship between phototype and cancer[39]. Once such a GPS is available, the association between the GPS and cancer can be identified as the association between the phototype and cancer.

Then, we tested the first criterium, i.e.: whether the GPS was associated with pigmentation variables and sun exposure; this was assessed in controls by multinomial logistic regression[40]. The results are presented as relative risk ratio (RRR) with 95% confidence intervals (CI). Note that the p-value, along with both the F statistic and R^2^, refer to the difference between the model including only the propensity score and the model including the pigmentation phenotype and the propensity score.

The second criterium (the GPS is independent from confounders) was tested by studying the association between prostate or BC risk factors and the GPS in controls. We categorized the GPS in quartiles and carried out statistical tests with chi-square or anova, according to the characteristics of the potential confounder.

The third criterium, i.e.: whether the GPS was NOT associated with cancer but via phototype was examined with MR-Egger method[39]. In order to do it, linear regression models were built with SNP–cancer association as Y variable and SNP–phenotype association as X variable, weighted by the inverse of variance.

The associations between the pigmentation phototype or GPS and BC and PCa were studied using logistic regression. To avoid bias linked to differences in case and control selection frequencies, each analysis was performed separately for BC and PCa and adjusted for a propensity score, which was designed including the recruiting center, the first 3 main principal components of genetic ancestry taken from genotyping data, age, education level, BMI the year before the tumor, and tobacco consumption five years before [41]. F statistics obtained as Chi square / (number of categories in the phenotype– 1) and provided an overall p-value for each model. Finally, as a sensitivity analysis, we constructed five different genetic pigmentation scores, one for each pigmentation trait (eye color, hair color, skin phototype, and the presence of freckles and tanning), from our data (S2 Table) we analyzed their relationship with cancer in the same way we have described for the GPS.

Results are reported as odds ratios (OR) with 95% confidence intervals (CI). All reported p-values are two-tailed. All statistical analyses were performed with the software Stata 14/SE (Stata Co., College Station, TX, US).

## Results

### Sample description and variables

1,738 BC cases and 1,910 controls (1,138 cases and 1,240 controls with genetic information) and 1,112 PCa cases and 1,493 controls (817 cases and 1,094 controls with genetic information) were included in the analysis; their main characteristics are reported in S3 Table. BC cases, compared to their controls, were 2.6 younger in average, the average age (SD) being 56.4 (12.6) years for cases and 59.0 (13.2) years for controls and their age at menopause was 48.8 years in both groups (SD 5.5 for cases and 5.3 for controls). There were no differences in BMI, tobacco consumption and education level. On the other hand, in the PCa collection, the average age was 66 years [(66.1 (7.3) for cases and 66.5 (8.6) for controls)]. PCa cases, compared to their controls, were 0.5 younger on average, and they had a lower education level with higher percentage of cases with primary level education only (39% vs. 32%) or less (23% vs. 19%). PCa cases and controls were similar in BMI and tobacco consumption.

### Pigmentation phototype and breast or prostate cancer

Skin, and hair color, and the presence of freckles response to the sun were not associated with the likelihood of developing BC or PCa (Table 1). However, having light brown or green eyes was associated with a 25% lower risk of BC (OR 0.75, 95% CI 0.61 to 0.91) and dark brown eyes a 21% lower risk of PCa (OR 0.79, CI 95%: 0.64 to 0.99), compared to having them dark brown.

**Table 1:** Relationship between phenotype and prostate and breast cancer.

		Prostate cancer								Breast cancer								
Phenotype	Category	Cases	Controls	OR	95% CI	p	F*	p*	R2(%)*	Cases	Controls	OR	95% CI	p		F*	p*	R2(%)*
Hair color	dark hair	919(83.1)	1067(83.4)	1			1.00	0.367	0.08	1301(75.6)	1292(74.2)	1				0.94	0.390	0.06
	Light brown hair	83(7.5)	99(7.7)	1.29	(0.90–1.85)	0.158				266(15.4)	301(17.3)	0.93	(0.73–1.17)		0.518			
	blonde hair	104(9.4)	113(8.8)	1.12	(0.79–1.57)	0.530				155(9.0)	149(8.6)	1.11	(0.81–1.53)		0.518			
Skin color	Dark skin	263(23.8)	306(21.1)	1			1.57	0.207	0.12	243(14.0)	277(14.7)	1				0.34	0.727	0.02
	Light brown skin	386(34.9)	542(37.3)	0.89	(0.72–1.12)	0.332				626(36.1)	681(36.2)	0.93	(0.77–1.13)		0.461			
	Fair skin	458(41.4)	604(41.6)	1.13	(0.88–1.46)	0.341				864(49.9)	922(49.0)	0.93	(0.70–1.24)		0.627			
Eye color	Black/dark brown	623(56.2)	742(51.1)	1			1.89	0.150	0.15	987(56.9)	967(51.4)	1				3.33	0.036	0.22
	Light brown/green	325(29.3)	473(32.6)	**0.79**	**(0.64–0.99)**	**0.040**				509(29.3)	622(33.1)	**0.75**	**(0.61–0.91)**		**0.005**			
	Blue/grey	161(14.5)	238(16.4)	0.91	(0.69–1.21)	0.533				239(13.8)	292(15.5)	1.09	(0.84–1.41)		0.531			
Freckles	No	185(16.7)	297(20.5)	1			5.44	0.066	0.31	472(27.2)	578(30.7)	1				3.19	0.074	0.11
	Yes	919(83.0)	1153(79.5)	1.27	(0.98–1.64)	0.070				1254(72.3)	1303(69.3)	1.09	(0.89–1.32)		0.410			
Behavior of the skin in the sun	I get tanned easily, I don't get burn	476(43.0)	607(42.2)	1			1.02	0.385	0.12	618(35.6)	657(35.0)	1				1.74	0.914	0.02
	I rarely get burned and then I get tanned	220(19.9)	289(20.1)	1.28	(0.98–1.67)	0.070				329(19.0)	377(20.1)	0.87	(0.67–1.11)		0.259			
	I get burned and then I get tanned	257(23.2)	344(23.9)	1.02	(0.80–1.31)	0.847				421(24.3)	461(24.6)	1.04	(0.83–1.31)		0.741			
	I get burned and almost never I get tanned	154(13.9)	198(13.8)	1.04	(0.78–1.40)	0.774				366(21.1)	382(20.4)	1.02	(0.80–1.31)		0.858			

Model adjusted for propensity score

*F statistics obtained as Chi square / (number of categories in the phenotype– 1). F, p and R2 refer to the comparison between the model with genetic score + propensity score and the model with only the propensity score

### Mendelian randomization, criterium 1: Association of genetic pigmentation score and phototype in controls

**Fig 1:**
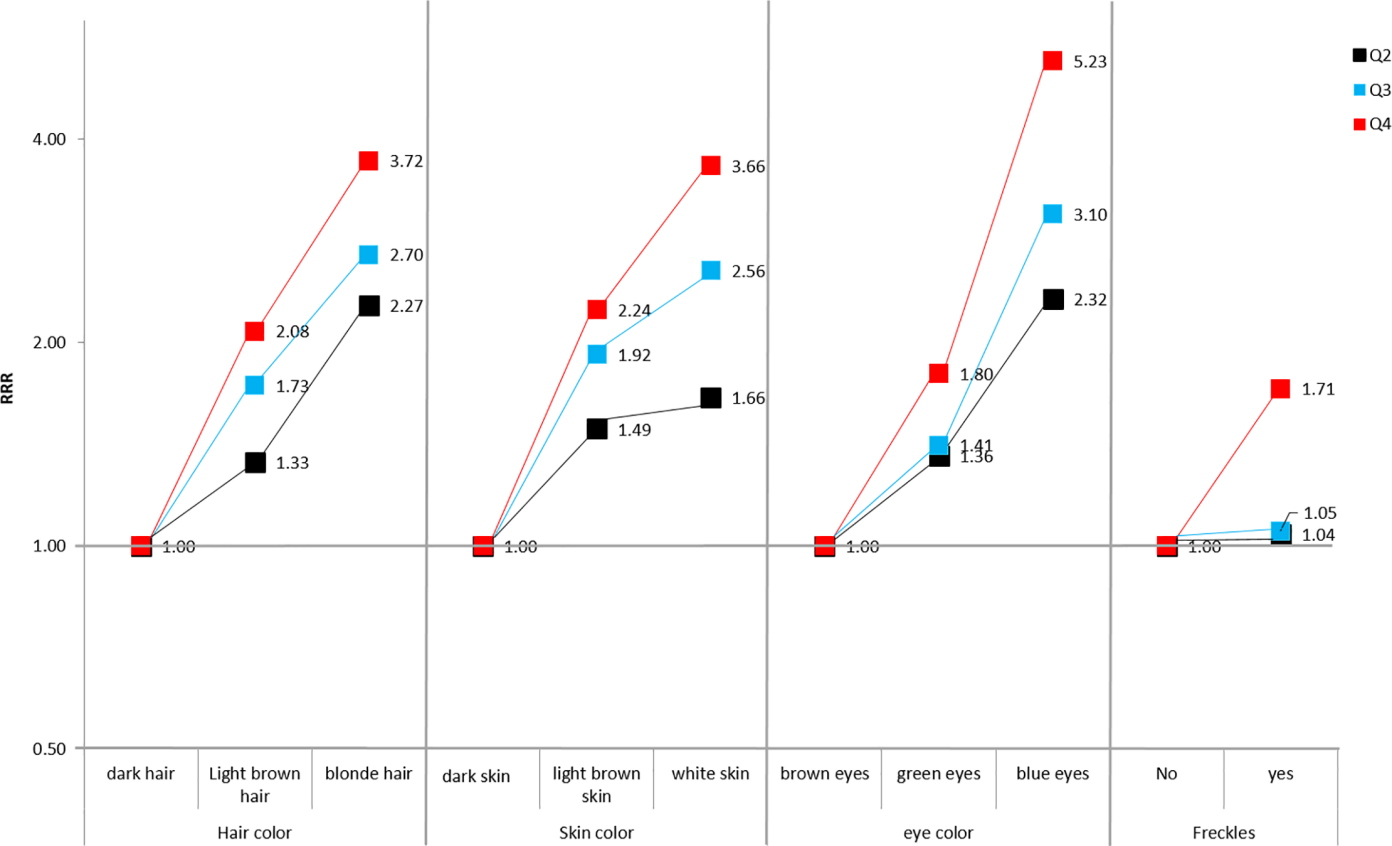
Relationship in controls, between phototype pigmentation and the genetic pigmentation score of the phototype in quartiles. Relative Risk Ratio (RRR).

Table 2 shows the association between the reported phototype and the GPS in controls for BC and PCa. Higher GPS was associated with fairer skin, blonde hair, blue eyes and presence of freckles. In addition, we could observe that when the GPS is divided into quartiles (Fig 1), the RRR pattern in any of these characteristics is similar: fixing the quartile of the GPS, RRR increases with fairer phototype, and fixing the phototype, RRR increases with higher GPS quartile. We carried out a sensitivity analysis using 5 specific genetic pigmentation scores for eye color, hair color, skin phototype, the presence of freckles and skin tanning; all five genetic pigmentation scores were developed using part of the 17 SNPs included in GPS. Each genetic pigmentation score was associated with its related phenotype as displayed in S4 Table. Thus, hair-color score explains a 3.7% of the variability in hair color while the eye-color score explains a 4.6% of the eye color. In the same way, higher hair-color score was associated with blonde hair (RRR 1.48 (1.35–1.62)) and higher eye-color score was associated with blue / grey (RRR 1.78 (1.63–1.94)).

**Table 2:** Relationship in controls between the genetic pigmentation score of the phototype (for each unit of increase) and the phototype. Relative Risk Ratio (RRR) and 95% Cis.

		RRR (95% CI) Genetic pigmentation score				
Phenotype	Category	N (%)	RRR (95% CI)	F*	p	R2(%)
Hair color	Dark hair	1779(78.62)	1 (reference)	9.09	<0.001	1.13
	Light brown hair	290(12.84)	1.12 (1.06–1.18)			
	Blonde hair	193(8.54)	1.20 (1.12–1.28)			
Skin color	Dark skin	365(15.76)	1 (reference)	16.58	<0.001	1.52
	Light brown skin	881(38.04)	1.16 (1.09–1.22)			
	Fair skin	1070(46.2)	1.27 (1.20–1.34)			
Eye color	Black/dark brown	1176(50.71)	1 (reference)	24.29	<0.001	2.20
	Light brown/green	792(34.15)	1.13 (1.08–1.18)			
	Blue/grey	351(15.14)	1.31 (1.23–1.38)			
Freckles	No	1723(74.36)	1 (reference)	15.63	<0.001	1.13
	Yes	594(25.64)	1.09 (1.04–1.14)			
Behavior of the skin in the sun	I get tanned easily, I don't get burn	902(38.98)	1 (reference)	8.89	<0.001	0.88
	I rarely get burned and then I get tanned	457(19.75)	1.11 (1.06–1.17)			
	I get burned and then I get tanned	560(24.20)	1.14 (1.08–1.19)			
	I get burned and almost never I get tanned	397(17.07)	1.22 (1.15–1.29)			

Model adjusted for propensity score

*F statistics obtained as Chi square / (number of categories in the phenotype– 1). F, p and R2 refer to the comparison between the model with genetic score + propensity score and the model with only the propensity score

### Mendelian randomization, criterium 2: The genetic pigmentation score is not associated with the confounders in the pigmentation–breast or prostate cancers relationships

S5 Table displays the results for the association between the GPS and risk factors for BC or PC showing a lack of such a relationship.

### Mendelian randomization, criterium 3: The genetic pigmentation score is not associated with cancer conditional on the phototype and confounders of the relationship between phototype and cancer

S6 Table shows the results of MR-Egger to test whether GPS is associated with cancer only through its association with the phototype; (i.e.: no horizontal pleiotropy). In this able, βrepresents an estimation of the phototype effect on cancer and α represents the bias that Mendelian randomization is introducing when estimating the phototype–cancer relationship (i.e.: the direct effect of GPS on cancer). Our results indicate that horizontal pleiotropy cannot be ruled out in skin color and skin behavior in the sun–PCa relationships, as α estimations differed from 0; the β estimates suggest that fairer skin and skins which burn or tan easily are associated with a lower risk of PCa, with odds ratios around 0.89. S1 Fig shows several plots of SNP effect on cancer vs SNP effect on the different phototype traits.

### Applying Mendelian randomization: Association between genetic pigmentation score and cancer

When we analyzed the association between GPS and cancer (Table 3), we verified that neither PCa nor BC were associated in the whole population. The same occurred when stratifying by age (<65 years / ≥65 years), BMI (<25 kg/m^2^ / ≥25 kg/m^2^) and menopausal status in women. As the clinical relevance of PCa with Gleason score < 7 is controversial, we reanalyzed the GPS–PCa association stratifying cases according to their Gleason score, again obtaining no association between GPS and PCa (results not shown). Also, when performing logistic regression analyses on specific genetic pigmentation scores and BC or PCa, the results indicated that none of these genetic scores was associated with PCa or BC.

**Table 3:** Association between genetic pigmentation scores of phototype and prostate and breast cancer.

		Prostate cancer			Breast cancer		
SCORES	Category	Cases/Controls	OR (95% CI)	p	Cases/Controls	OR (95% CI)	p
**GPS**	**All**	**813/1084**	**1.01 (0.97–1.05)**	**0.706**	**1133/1236**	**0.99 (0.96–1.03)**	**0.637**
** **	**< 65 years**	**341/428**	**1.05 (0.99–1.12)**	**0.129**	**820/766**	**0.99 (0.95–1.03)**	**0.621**
** **	**≥65 years**	**472/656**	**0.97 (0.92–1.03)**	**0.343**	**313/470**	**1.00 (0.94–1.08)**	**0.909**
** **	**BMI < 25 Kg/m2**	**205/256**	**0.96 (0.88–1.05)**	**0.392**	**511/577**	**0.98 (0.93–1.04)**	**0.513**
** **	**BMI ≥25 Kg/m2**	**596/803**	**1.03 (0.98–1.07)**	**0.297**	**551/537**	**1.00 (0.94–1.05)**	**0.865**
** **	**premenopausal**				**403/361**	**0.98 (0.92–1.05)**	**0.524**
** **	**postmenopausal**	** **	** **	** **	**729/875**	**0.99 (0.95–1.04)**	**0.825**
**Hair color score**	**All**	816/1092	1.01 (0.96–1.06)	0.753	1138/1240	0.97 (0.93–1.02)	0.265
** **	**< 65 years**	341/431	1.02 (0.94–1.10)	0.709	825/769	0.96 (0.91–1.02)	0.171
** **	**≥65 years**	475/661	1.00 (0.94–1.07)	0.995	313/471	1.00 (0.92–1.09)	0.974
** **	**BMI < 25 Kg/m2**	206/261	0.96 (0.87–1.07)	0.465	513/579	0.98 (0.92–1.04)	0.481
** **	**BMI ≥25 Kg/m2**	598/806	1.02 (0.97–1.08)	0.458	554/539	0.97 (0.91–1.04)	0.429
** **	**premenopausal**				404/362	0.99 (0.92–1.08)	0.890
** **	**postmenopausal**				733/878	0.96 (0.91–1.02)	0.199
**Skin color score**	**All**	815/1089	1.00 (0.95–1.05)	0.94	1136/1237	1.00 (0.96–1.05)	0.901
** **	**< 65 years**	341/430	1.02 (0.95–1.10)	0.621	823/767	1.01 (0.95–1.06)	0.820
** **	**≥65 years**	474/659	0.98 (0.92–1.05)	0.558	313/470	1.01 (0.92–1.10)	0.882
** **	**BMI < 25 Kg/m2**	206/260	0.92 (0.83–1.02)	0.109	513/578	0.99 (0.93–1.06)	0.851
** **	**BMI ≥25 Kg/m2**	597/804	1.03 (0.98–1.09)	0.262	552/537	1.01 (0.95–1.08)	0.702
** **	**premenopausal**				404/361	1.02 (0.94–1.10)	0.687
** **	**postmenopausal**				731/876	0.99 (0.94–1.05)	0.831
**Eye color score**	**All**	816/1091	1.01 (0.95–1.07)	0.741	1136/1240	1.01 (0.96–1.07)	0.574
** **	**< 65 years**	341/431	1.05 (0.96–1.15)	0.271	823/769	1.00 (0.94–1.07)	0.924
** **	**≥65 years**	475/660	0.98 (0.91–1.05)	0.584	313/471	1.05 (0.95–1.15)	0.344
** **	**BMI < 25 Kg/m2**	206/260	1.00 (0.89–1.12)	0.993	512/579	1.00 (0.93–1.08)	0.957
** **	**BMI ≥25 Kg/m2**	598/806	1.02 (0.95–1.08)	0.623	553/539	1.03 (0.95–1.10)	0.488
** **	**premenopausal**				404/362	1.01 (0.92–1.11)	0.815
** **	**postmenopausal**				731/878	1.01 (0.95–1.08)	0.658
**Freckles score**	**All**	814/1087	1.00 (0.93–1.08)	0.983	1135/1239	1.02 (0.96–1.09)	0.514
** **	**< 65 years**	341/428	0.98 (0.88–1.10)	0.784	822/768	1.04 (0.96–1.12)	0.325
** **	**≥65 years**	473/659	1.00 (0.91–1.11)	0.925	313/471	0.99 (0.88–1.12)	0.921
** **	**BMI < 25 Kg/m2**	205/256	0.94 (0.81–1.10)	0.433	511/578	0.99 (0.91–1.09)	0.91
** **	**BMI ≥25 Kg/m2**	597/806	1.03 (0.95–1.12)	0.481	553/539	1.05 (0.95–1.15)	0.339
** **	**premenopausal**				403/362	1.01 (0.91–1.13)	0.799
** **	**postmenopausal**				731/877	1.03 (0.95–1.11)	0.526
**Tanning score**	**All**	1108/1484	1.01 (0.97–1.06)	0.596	1736/1906	0.99 (0.95–1.03)	0.684
** **	**< 65 years**	461/567	1.04 (0.97–1.12)	0.29	1269/1170	0.99 (0.94–1.04)	0.763
** **	**≥65 years**	647/917	0.98 (0.92–1.05)	0.604	467/736	1.00 (0.92–1.08)	0.932
** **	**BMI < 25 Kg/m2**	279/341	0.93 (0.84–1.02)	0.136	772/847	0.98 (0.93–1.05)	0.604
** **	**BMI ≥25 Kg/m2**	810/1002	1.05 (0.99–1.11)	0.109	834/796	1.00 (0.94–1.06)	0.932
** **	**premenopausal**				611/547	1.00 (0.93–1.08)	0.954
** **	**postmenopausal**				1124/1353	0.99 (0.94–1.04)	0.628

Model adjusted for propensity score

## Discussion

In our study, the advantages of the method of Mendelian randomization were used to verify whether there was a causal relationship between phototype (eye color, hair color, skin phototype, and the presence of freckles) and the risk of BC and PCa. We found that phototype was not associated with BC or PCa. Mendelian randomization uses genetic markers as instrumental variables or proxies for the exposure (phototype). Thus, our genetic marker was a genetic score combining alleles which have been described previously as associated with phototype (hair, skin, eyes and freckles); according to our analysis, this genetic score was not associated with the risk of BC or PCa.

To the best our knowledge, only two studies have directly examined the relationship between the exposure of pigmentation phenotypes (eye color, hair, skin and the presence of freckles as the way in which exposure to the phenotype was measured) and the incidence of PCa [26,27]; however, the epidemiological evidence is still inconsistent. Results found in an observational study may be due to confounding factors associated with fair skin phototype and cancer simultaneously. A number of putative confounders have been identified, namely exposure to UV rays, seasons of the year; use of high factor sun cream, level of vitamin D or estrogen levels, could be mediators in such a relationship [42,43]. For example, a possible protective effect of exposure to UV on PCa was hypothesized to be mediated through the increase of vitamin D level resulting from the synthesis of vitamin D epidermal induced by sunlight[43,44]. On the other hand, some studies have shown an inverse association between vitamin D levels and prostate size[45,46] and meta-analyses have shown an association between prostate[46] and breast[47] cancer and vitamin D. The usual ways for controlling confounders in observational studies include multivariate regression, stratified analysis and other; unfortunately, no method guarantees the absence of residual confounding.

Information bias could also have influenced results from observational studies, since most studies reporting associations between phototype and cancer have used data reported by the participants through an interview (although these variables may very well be reported accurately, however, there could be some subjectivity in the answer too) about hair, skin and eyes color, instead of objectively measured data. A prior study on sun exposure and PCa risk, described darker pigmentation on the forehead (measured using a reflectometer), was associated with reduced PCa risk [48]. Conversely, other studies have observed that lower sun exposure was associated with an increased risk of PCa [43,44,49]. A reason for this may be that people who are sun -sensitive avoid and / or protect themselves with clothing and sun cream to prevent sunburn and skin cancer.

On the other hand, reverse causality may also partly explain the association between phototype and cancer reported in retrospective or cross-sectional studies. We have also identified two studies that directly examined the relationship between pigmentation phenotypes and the incidence of PCa, another hormone-dependent tumor and their results were somewhat contradictory: On the one hand, Bonilla et al found that British white men who were more likely to have freckles, a lighter skin color and were more prone to burning than tanning had a higher risk of developing PCa than men without such variant[26]; on the other hand, Weinstein et al. paper found no association between eye color and skin phototype[27], but red-haired participants in the study were significantly less likely to develop PCa than men with light brown hair[27]. However, it is unclear whether these associations are causal; in fact, the findings may be due to confounders simultaneously associated with the fair skin phototype and cancer, such as ultraviolet radiation (UV) exposure, seasons of the year, level of vitamin D or use of sun cream with high protection. As these potential confounders may act in different directions, one cannot discard that different composition of the studied populations could have dealt to such a contradictory results.

### Mendelian randomization

Mendelian randomization allows the researchers to control for known and unknown confounding factors if these factors are not associated with the genetic instruments and to eliminate information bias, overcoming the problems that are frequent in strictly observational studies. Firstly, genotypes are determined at conception and remain constant throughout life; they are randomly distributed in conception in relation to potential confounding factors. Secondly, Mendelian randomization is not influenced by reverse causation, since BC or PCa cannot change the germline genotype of an individual. If the phototype is truly a causal risk factor in cancer development, it would be expected that genetic variants associated with the phototype will also increase cancer risk.

The GPS we have created was not derived from our data as the SNPs were selected from the literature. It fulfills all three criteria for Mendelian randomization: Firstly, it is associated with phototype in controls Secondly, the GPS was not associated with known risk factors for prostate or BC, which would have been confounders in the phototype–cancer relationship; of note, this way of testing cannot rule out associations with unmeasured confounders. The third Mendelian randomization assumption–i.e.: the GPS is not associated with cancer conditioned to phototype and confounders (= no pleiotropy)- cannot be directly tested with data. In this regard, MR-Egger regression could be considered a sensitivity analysis as it allows for estimation of the bias introduced by a putative direct effect (α in S6 Table) and once such bias was removed (β in S6 Table), the true phototype–cancer link was apparent. According to MR-Egger results, the possibility of pleiotropy can be excluded for hair color, eye color and freckles–cancer relationship, but not for skin color and skin behavior in the sun–PCa relationships. In this regard, MR-Egger suggested that fair skin and skins getting easily burned or tanned were associated with lower risk of PCa; the putative effects suggested by MR-Egger regression, however, seem to be unimportant and require further confirmation.

As far as we know, there is only one article that uses three predictive genetic pigmentation scores associated with phototypes[27] (6 SNPs used for building a skin color score, 13 for a tanning score and 8 SNPs for a freckling score). We have created five genetic pigmentation scores one for each characteristic of the phototype and another global one that agglutinates the whole phototype, improving the predictive capacity of Bonilla et al.[27]. Bonilla et al also observed that individuals who had a tendency to burn and spent less time sunbathing, had less vitamin D level in plasma and a greater susceptibility to PCa. However, in our research, using a global score with the 17 SNPs published in the literature up to January 2017, we have failed to find that association with PCa. This negative result was confirmed in a sensitivity analysis of ours using phototype-specific genetic pigmentation scores.

### Limitations

Our study has some limitations. Firstly, genotyping was available only for 65% of our patients. Secondly, the sample size for PCa in our study is relatively small when compared with a previous Mendelian randomization study (1136 cases in Bonilla et al [27] vs. 817cases in our analysis); therefore, our study could have little power to detect small causal effects in BC and PCa if they exist; also, chance could still play a role in the results. Thirdly, our data proceed from a case-control study, so self-selection -especially in controls- cannot be ruled out; a putative mechanism for self-selection could be that people with higher education would be more prone to participate and would have fairer phototype; however, controls scarcely displayed such association between phototype and education level, with Goodman-Kruskal gamma = 0.02 in men and 0.03 in women, which makes it difficult to be a source of bias. In fifth place, we have selected only participants of Caucasian origin; Spain has largely been a country to emigrate from, although this trend changed around 1995, when Spain begun to receive immigrants mainly from North Africa, South America and Eastern Europe; most of them were young adults, which makes them unlikely to be selected for our study. This selection could limit the generalization of our results.

In conclusion, our results demonstrate that hormone-dependent breast and prostate cancers are not associated with eye color, hair color, skin phototype, and the presence of freckles. It is also shown that genetic pigmentation scores can be used as instrumental variables for pigmentation to avoid confounding and problems related to information reliability.

## Supporting information

S1 Table List of 17SNPs selected and the genomic region and gene they belong to, according to the risk allele identified in the literature. (DOCX)

S2 Table SNPs included in the different scores according to the risk allele as identified in our study. (DOCX)

S3 Table Sample description. (DOCX)

S4 Table Relationship in controls, between different genetics scores of the phototype (for each unit of increase) and the phototype. Relative Risk Ratio (RRR) and 95% CIs.
(DOCX)

S5 Table Association between risk factors of prostate and breast cancer and the genetic score in controls. (DOCX)

S6 Table Results of MR-Egger regression. (DOCX)

S1 Fig MR-Egger regression: Plots of SNP effect on cancer vs SNP effect on the different phototype traits. (TIF)

## Abbreviations

BC: breast cancer
PCa: prostate cancer
BMI: body mass index
UV: ultraviolet radiation
GWAS: genome-wide association studies
HWE: Hardy-Weinberg equilibrium
MCC-Spain: Multicase-control Spain
GPS: genetic pigmentation score
OR: Odds ratios
RRR: relative risk ratio
CI: confidence intervals
