# Supplementary material for: Pigmentation phototype and prostate and breast cancer in a select Spanish population—A Mendelian randomization analysis in the MCC-Spain study
Source: PLoS One. 2018 Aug 14;13(8):e0201750. doi: 10.1371/journal.pone.0201750 (PMC6091948; doi:10.1371/journal.pone.0201750)
Supplement: S1 Table — (DOCX) [file pone.0201750.s001.docx]

**S1 Table. List of 17SNPs selected and the genomic region and gene they belong to, according to the risk allele identified in the literature.**

| **Number** | **SNPS** | **CHR_ID** | **REGION** | **CHR POS** | **MAPPED GENE** | **CONTEXT** | **DISEASE TRAIT** | **PUBMED *** | **STRONGEST SNP RISK ALLELE** | **RISK ALLELE FREQUENCY** | **P VALUE** | **OR** | **[95 CI]** | **LD with** |
| --- | --- | --- | --- | --- | --- | --- | --- | --- | --- | --- | --- | --- | --- | --- |
|  |  |  |  |  |  |  |  |  |  |  |  | Published* |  |  |
| 1 | rs1003719 | 21 | 21q22.13 | 37118795 | TTC3 | intron_variant | Eye color traits | 20463881 | A | NR | 2.00E-10 | 0.9 | [NR] |  |
| 2 | rs1015362 | 20 | 20q11.22 | 34150806 | RPS2P1 - ASIP | regulatory_region_variant | Red vs. non-red hair color | 18488028 | G | 0.08 | 3.00E-09 | 1.76 | [1.34-2.31] |  |
|  |  |  |  |  |  |  | Burning and freckling | 18488028 | G | 0.08 | 6.00E-37 | 2.56 | [2.06-3.18] |  |
|  |  |  |  |  |  |  | Freckles | 18488028 | G | 0.08 | 8.00E-29 | 1.95 | [1.65-2.32] |  |
|  |  |  |  |  |  |  | Skin sensitivity to sun | 18488028 | G | 0.08 | 2.00E-24 | 1.76 | [1.49-2.08] |  |
| 3 | rs1042602 | 11 | 11q14.3 | 89178528 | TYR, LOC107984363 | missense_variant | Skin pigmentation | 17999355 | A | 0.16 | 4.00E-10 | 0.23 | [0.14-0.38] |  |
|  |  |  |  |  |  |  | Freckles | 17952075 | C | 0.67 | 2.00E-11 | 1.32 | [1.17-1.49] |  |
| 4 | rs12203592 | 6 | 6p25.3 | 396321 | IRF4 | intron_variant | Black vs. blond hair color | 18483556 | T | NR | 7.00E-127 | 0.70 | [0.69-0.72] |  |
|  |  |  |  |  |  |  | Black vs. red hair color | 18483556 | T | NR | 9.00E-28 | 0.73 | [0.70-0.78] |  |
|  |  |  |  |  |  |  | Hair color | 20585627 | T | 0.18 | 2.00E-28 | 1.80 | [NR] |  |
|  |  |  |  |  |  |  | Eye color | 20585627 | T | 0.18 | 2.00E-15 | 0.66 | [NR] |  |
|  |  |  |  |  |  |  | Freckling | 20585627 | T | 0.18 | 2.00E-91 | 5.00 | [NR] |  |
|  |  |  |  |  |  |  | Tanning | 23548203 | T | NR | 3.00E-23 | 1.42 | [1.34-1.51] |  |
|  |  |  |  |  |  |  | Hair color | 23548203 | T | NR | 1.00E-28 | 0.70 | [0.66-0.75] |  |
|  |  |  |  |  |  |  | Sunburns | 23548203 | T | NR | 2.00E-06 | 3.10 | [1.93-4.95] |  |
|  |  |  |  |  |  |  | Skin colour saturation | 25963972 | T | 0.09 | 3.00E-09 | 0.99 | [NR] |  |
|  |  |  |  |  |  |  | Facial pigmentation | 25705849 | T | 0.09 | 2.00E-27 | 1.28 | [NR] |  |
|  |  |  |  |  |  |  | Hair greying | 26926045 | T | NR | 3.00E-13 |  |  |  |
|  |  |  |  |  |  |  | Hair color | 26926045 | T | NR | 1.00E-13 |  |  |  |
|  |  |  |  |  |  |  | Monobrown | 27182965 | T | NR | 1.00E-10 | 1.03 | [1.02-1.04] |  |
| 5 | rs12210050 | 6 | 6p25.3 | 475489 | LOC105374875 | intergenic_variant | Tanning | 19340012 | T | NR | 5.00E-14 | 1.25 | [1.17-1.32] |  |
| 6 | rs12896399 | 14 | 14q32.12 | 92307319 | LOC105370627 | intergenic_variant | Blond vs. brown hair color | 17952075 | T | 0.44 | 1.00E-48 | 2.56 | [2.12-3.09] |  |
|  |  |  |  |  |  |  | Blue vs. green eyes | 17952075 | T | 0.40 | 4.00E-38 | 2.06 | [1.76-2.42] |  |
|  |  |  |  |  |  |  | Black vs. blond hair color | 18483556 | T | NR | 8.00E-21 | 0.83 | [0.80-0.87] |  |
|  |  |  |  |  |  |  | Hair color | 20585627 | T | 0.56 | 5.00E-13 | 1.36 | [NR] |  |
|  |  |  |  |  |  |  | Eye color | 20585627 | T | 0.56 | 1.00E-16 | 1.41 | [NR] |  |
|  |  |  |  |  |  |  | Eye color | 20585627 | T | 0.56 | 2.00E-23 | 1.73 | [NR] |  |
|  |  |  |  |  |  |  | Hair color | 23548203 | T | NR | 1.50E-36 | 1.17 | [1.15-1.20] |  |
|  |  |  |  |  |  |  | Eye color | 23548203 | T | NR | 4.10E-11 | 0.72 | [0.65-0.79] |  |
|  |  |  |  |  |  |  | ~~Eye color~~ | ~~23548203~~ | ~~G~~ | ~~NR~~ | ~~3.00E-07~~ | ~~0.27~~ | ~~[0.17-0.37]~~ |  |
| 7 | rs1393350 | 11 | 11q14.3 | 89277878 | TYR, LOC107984363 | intron_variant | Blue vs. green eyes | 17952075 | A | NR | 3.30E-12 | 1.52 | [1.28-1.81] |  |
|  |  |  |  |  |  |  | Skin sensitivity to sun | 17952075 | A | NR | 1.60E-06 | 1.26 | [1.11-1.43] |  |
|  |  |  |  |  |  |  | Tanning | 19340012 | A | NR | 2.00E-13 | 1.21 | [1.14-1.28] |  |
|  |  |  |  |  |  |  | Eye color | 20585627 | A | 0.73 | 3.00E-09 | 1.32 | [NR] |  |
| 8 | rs17094273 | 14 | 14q32.2 | 96637470 | PAPOLA - RN7SKP108 | downstream_gene_variant | Tanning | 19340012 | A | NR | 9.00E-08 | 1.22 | [1.13-1.32] |  |
| 9 | rs1800407 | 15 | 15q13.1 | 27985172 | OCA2 | missense_variant | eye color | 4947861 | C | 0.93 | NR | NR | [NR] |  |
| 10 | rs1805007 | 16 | 16q24.3 | 89919709 | MC1R | missense_variant | Freckles | 17952075 | T | 0.142 | 1.00E-96 | 4.37 | [3.56-5.37] |  |
|  |  |  |  |  |  |  | Blond vs. brown hair color | 17952075 | T | 0.142 | 2.00E-13 | 2.34 | [1.69-3.24] |  |
|  |  |  |  |  |  |  | Red vs non-red hair color | 17952075 | T | 0.142 | 2.00E-142 | 12.47 | [9.37-16.60] |  |
|  |  |  |  |  |  |  | Skin sensitivity to sun | 17952075 | T | 0.142 | 2.00E-55 | 2.94 | [2.42-3.58] |  |
|  |  |  |  |  |  |  | Tanning | 23548203 | T | NR | 1.00E-65 | 1.47 | [1.42-1.54] |  |
|  |  |  |  |  |  |  | Hair color | 23548203 | T | NR | 3.00E-09 | 1.17 | [1.11-1.25] |  |
|  |  |  |  |  |  |  | Sunburns | 23548203 | T | NR | 1.50E-19 | 0.19 | [0.13-0.27] |  |
|  |  |  |  |  |  |  | Perceived skin darkness | 25963972 | T | NR | NR | NR | [NR] |  |
| 11 | rs1805008 | 16 | 16q24.3 | 89919736 | MC1R | missense_variant | Skin colour saturation | 25963972 | T | NR | NR | NR | [NR] |  |
|  |  |  |  |  |  |  | Skin colour saturation | 25963972 | T | NR | NR | NR | [NR] |  |
| 12 | rs2153271 | 9 | 9p22.2 | 16864523 | BNC2 | intron_variant | Freckling | 20585627 | T | 0.41 | 4.00E-10 | 0.67 | [NR] |  |
| 13 | rs4778138 (also called rs11855019) | 15 | 15q13.1 | 28090674 | OCA2 | intron_variant | Skin/hair/eye pigmentation | 18483556 | G | 0.16 | 2.10E-24 | 0.75 | [0.71-0.80] |  |
| 14 | rs619865 | 20 | 20q11.22 | 35279894 | EIF6 | intron_variant | Freckling | 20585627 | A | 0.1 | 5.00E-14 | 2.16 | [NR] |  |
| 15 | rs7279297 | 21 | 21q22.3 | 41807559 | PRDM15 | intron_variant | Tanning | 19340012 | A | NR | 3.00E-06 | 1.12 | [1.06-1.20] |  |
| 16 | rs916977 | 15 | 15q13.1 | 28268218 | HERC2 | intron_variant | Iris color | 18252221 | C | 0.13 | 1.00E-43 | NR | [NR] |  |
| 17 | rs9894429 | 17 | 17q25.3 | 81629785 | NPLOC4 | synonymous_variant | Eye color traits | 20463881 | C | NR | 9.00E-14 | 0.99 | [NR] |  |
| Removed | rs12931267 | 16 | 16q24.3 | 89752324 | FANCA | intron_variant | Hair color | 20585627 | G | 0.08 | 3.00E-10 | 0.57 | [NR] | rs1805007 |
|  |  |  |  |  |  |  | Hair color | 20585627 | G | 0.08 | 5.00E-87 | 1.75 | [NR] |  |
|  |  |  |  |  |  |  | Freckling | 20585627 | G | 0.08 | 8.00E-62 | 6.55 | [NR] |  |
|  |  |  |  |  |  |  | Skin sensitivity to sun | 25963972 | G | 0.091 | 8.00E-23 | 0.64 | [0.59-0.70] |  |

LD: linkage disequilibrium. All SNPs with LD >0.8 were removed. OR: Odds Ratio. PMID: PubMed ID of the article the OR was published.

OR Published and PMID Obtained from the GWAS Catalog
