# Supplementary material for: Pigmentation phototype and prostate and breast cancer in a select Spanish population—A Mendelian randomization analysis in the MCC-Spain study
Source: PLoS One. 2018 Aug 14;13(8):e0201750. doi: 10.1371/journal.pone.0201750 (PMC6091948; doi:10.1371/journal.pone.0201750)
Supplement: S2 Table — (DOCX) [file pone.0201750.s002.docx]

**S2 Table. SNPs included in the different scores according to the risk allele as identified in our study.**

|  | | **Risk allele associated with different score** | | | | |
| --- | --- | --- | --- | --- | --- | --- |
| **SNP** | **Major/minor alleles** | **Hair color score** | **Skin color score** | **Eye color score** | **Freckles score** | **Tanning score** |
| **rs1003719** | G/A |  | **G** |  |  |  |
| **rs1015362** | C/T |  |  | *C* |  |  |
| **rs1042602** | C/A | **C** | **C** |  |  | **C** |
| **rs12203592** | C/T | ***C*** | **C** | **C** | **C** | **C** |
| **rs12210050** | C/T |  |  |  | **C** | **C** |
| **rs12896399** | G/T | **G** |  | **G** |  |  |
| **rs1393350** | G/A |  |  | **G** | **G** |  |
| **rs17094273** | G/A |  |  | **G** |  | **G** |
| **rs1800407** | C/T |  | **C** |  |  | **C** |
| **rs1805007** | C/T | **C** | **C** |  | **C** | **C** |
| **rs1805008** | C/T |  | **C** |  |  | **C** |
| **rs2153271** | T/C | *T* | *T* |  | T | T |
| **rs4778138** | A/G | *A* | *A* | *A* |  | *A* |
| **rs619865** | G/A | **G** | **G** |  | **G** | **G** |
| **rs7279297** | A/G |  | *A* |  |  | *A* |
| **rs916977** | C/T | *C* | *C* | *C* |  | *C* |
| **rs9894429** | C/T | *C* |  |  |  |  |

*In bold the associated risk allele and in gray box, the risk allele when the protective allele has been substituted by it*
