# Supplementary material for: Pigmentation phototype and prostate and breast cancer in a select Spanish population—A Mendelian randomization analysis in the MCC-Spain study
Source: PLoS One. 2018 Aug 14;13(8):e0201750. doi: 10.1371/journal.pone.0201750 (PMC6091948; doi:10.1371/journal.pone.0201750)
Supplement: S3 Table — (DOCX) [file pone.0201750.s003.docx]

**S3 Table. Sample description.**

|  |  | **Prostate cancer** | | | **Breast cancer** | | |
| --- | --- | --- | --- | --- | --- | --- | --- |
| **Variable** | **Category** | **Cases (%)** | **Controls (%)** | **p** | **Cases (%)** | **Controls (%)** | **p** |
| **Age** | **<45 years** | 1(0) | 20(1) | <0.001 | 352(20) | 344(18) | <0.001 |
|  | **45 – 54 years** | 70(6) | 112(8) |  | 456(26) | 413(22) |  |
|  | **55 – 64 years** | 391(35) | 438(29) |  | 463(27) | 416(22) |  |
|  | **65 – 74 years** | 495(45) | 633(42) |  | 289(17) | 446(23) |  |
|  | **≥75 years** | 155(14) | 290(19) |  | 178(10) | 291(15) |  |
| **Educational level** | **Less than primary school** | 261(23) | 289(19) | <0.001 | 270(16) | 329(17) | 0.10 |
|  | **Primary school** | 439(39) | 485(32) |  | 563(32) | 586(31) |  |
|  | **Secondary school** | 242(22) | 405(27) |  | 574(33) | 588(31) |  |
|  | **University** | 170(15) | 314(21) |  | 331(19) | 407(21) |  |
| **Menopausal status** | **Premenopausal** |  |  |  | 612(35) | 548(29) | <0.001 |
|  | **Postmenopausal** |  |  |  | 1125(65) | 1356(71) |  |
| **Smoking** | **Never smoker** | 328(30) | 397(27) | 0.09 | 970(56) | 1141(60) | 0.07 |
|  | **Former smoker** | 458(41) | 678(46) |  | 286(17) | 296(16) |  |
|  | **Current smoker** | 319(29) | 413(28) |  | 469(27) | 465(24) |  |
| **Body Mass Index (kg/m^2^)** | **<18.5** | 2(0) | 8(1) | 0.46 | 28(1.7) | 38(2) | 0.18 |
|  | **18.5 – 24.9** | 279(26) | 337(25) |  | 745(46) | 811(49) |  |
|  | **25.0 – 29.9** | 558(51) | 687(51) |  | 542(34) | 509(30) |  |
|  | **≥30.0** | 254(23) | 320(24) |  | 293(18) | 289(18) |  |
