# Supplementary material for: Pigmentation phototype and prostate and breast cancer in a select Spanish population—A Mendelian randomization analysis in the MCC-Spain study
Source: PLoS One. 2018 Aug 14;13(8):e0201750. doi: 10.1371/journal.pone.0201750 (PMC6091948; doi:10.1371/journal.pone.0201750)
Supplement: S4 Table — Relative Risk Ratio (RRR) and 95% CIs. (DOCX) [file pone.0201750.s004.docx]

**S4 Table. Relationship in controls, between different genetics scores of the phototype (for each unit of increase) and the phototype. Relative Risk Ratio (RRR) and 95% Cis.**

|  |  |  | Hair color score | | | |
| --- | --- | --- | --- | --- | --- | --- |
| Phenotype | Category | N (%) | RRR (95% CI) | F* | p | R2(%) |
| Hair color | Dark hair | 1784(78.56) | 1 (reference) | 30.20 | <0.001 | 3.7 |
|  | Light brown hair | 293(12.90) | 1.21 (1.12-1.30) |  |  |  |
|  | Blonde hair | 194(8.54) | 1.48 (1.35-1.62) |  |  |  |
|  |  |  |  |  |  |  |
|  |  |  | skin color score | | | |
|  |  | N (%) | RRR (95% CI) | F* | p | R2(%) |
| Skin color | Dark skin | 365(15.72) | 1 (reference) | 19.53 | <0.001 | 1.8 |
|  | Light brown skin | 884(38.07) | 1.20 (1.13-1.29) |  |  |  |
|  | Fair skin | 1073(46.21) | 1.33 (1.25-1.43) |  |  |  |
|  |  |  |  |  |  |  |
|  |  |  | eyes color score | | | |
|  |  | N (%) | RRR (95% CI) | F* | p | R2(%) |
| Eye color | Black/dark brown | 1177(50.52) | 1 (reference) | 50.64 | <0.001 | 4.6 |
|  | Light brown/green | 799(34.29) | 1.26 (1.18-1.33) |  |  |  |
|  | Blue/grey | 354(15.19) | 1.78 (1.63-1.94) |  |  |  |
|  |  |  |  |  |  |  |
|  |  |  | freckles score | | | |
|  |  | N (%) | RRR (95% CI) | F* | p | R2(%) |
| Freckles | No | 1727(77.34) | 1 (reference) | 29.25 | <0.001 | 2.1 |
|  | Yes | 596(25.66) | 1.32 (1.23-1.42) |  |  |  |
|  |  |  |  |  |  |  |
|  |  |  | tanning score | | | |
|  |  | N (%) | RRR (95% CI) | F* | p | R2(%) |
| Behavior of the skin in the sun | I get tanned easily, I don't get burn | 1260(38.15) | 1 (reference) | 13.38 | <0.001 | 1.3 |
|  | I rarely get burned and then I get tanned | 663(20.07) | 1.17 (1.10-1.24) |  |  |  |
|  | I get burned and then I get tanned | 802(24.28) | 1.21 (1.15-1.29) |  |  |  |
|  | I get burned and almost never I get tanned | 578(17.50) | 1.31 (1.23-1.40) |  |  |  |

Model adjusted for propensity score

*F statistics obtained as Chi square / (number of categories in the phenotype – 1)

F, p and R2 refer to the comparison between the model with genetic score + propensity score and the model with only the propensity score
