# Supplementary material for: Pigmentation phototype and prostate and breast cancer in a select Spanish population—A Mendelian randomization analysis in the MCC-Spain study
Source: PLoS One. 2018 Aug 14;13(8):e0201750. doi: 10.1371/journal.pone.0201750 (PMC6091948; doi:10.1371/journal.pone.0201750)
Supplement: S5 Table — (DOCX) [file pone.0201750.s005.docx]

**S5 Table. Association between risk factors of prostate and breast cancer and the genetic score in controls.**

|  |  | **Genetic Score** | | | | | |  |
| --- | --- | --- | --- | --- | --- | --- | --- | --- |
|  |  | **Q1** | **Q2** | **Q3** | | **Q4** | |  |
| **Risk factors for prostate cancer** | **Category** | **N(%)** | **N(%)** | **N(%)** | | **N(%)** | | **p** |
| **Age (years)** | **mean(sd)** | 66.17(0.46) | 66.62(0.44) | 66.76(0.68) | | 66.50(0.54) | | 0.870 |
| **Ethnicity or race** | **White /caucasian** | 328(100) | 365(100) | 152(100) | | 239(100) | |  |
|  | **Maghreb** | 0(0) | 0(0) | 0(0) | | 0(0) | |  |
|  | **Another African** | 0(0) | 0(0) | 0(0) | | 0(0) | |  |
|  | **Asian** | 0(0) | 0(0) | 0(0) | | 0(0) | |  |
|  | **Gypsy** | 0(0) | 0(0) | 0(0) | | 0(0) | |  |
|  | **Other** | 0(0) | 0(0) | 0(0) | | 0(0) | |  |
| **Education** | **Less than primary school** | 344(21.5) | 402(21.2) | 202(23.7) | | 290(21.3) | | 0.720 |
|  | **Primary school** | 598(37.4) | 683(35.9) | 294(34.5) | | 487(35.7) | |  |
|  | **Secondary school** | 413(25.8) | 499(26.3) | 209(24.5) | | 357(26.2) | |  |
|  | **University** | 243(15.2) | 316(16.6) | 147(17.3) | | 230(16.9) | |  |
| **Recruitment province** | **Madrid** | 313(19.6) | 394(20.7) | 156(18.3) | | 251(18.4) | | 0.018 |
|  | **Barcelona** | 378(23.7) | 445(23.4) | 200(23.5) | | 336(24.6) | |  |
|  | **Navarra** | 92(5.8) | 136(7.2) | 56(6.6) | | 98(7.2) | |  |
|  | **Guipuzcoa** | 151(9.4) | 195(10.3) | 97(11.4) | | 175(12.8) | |  |
|  | **Leon** | 276(17.3) | 303(15.9) | 130(15.3) | | 165(12.1) | |  |
|  | **Asturias** | 67(4.2) | 98(5.2) | 46(5.4) | | 90(6.6) | |  |
|  | **Huelva** | 8(0.5) | 16(0.8) | 9(1.1) | | 15(1.1) | |  |
|  | **Cantabria** | 202(12.6) | 200(10.5) | 101(11.9) | | 149(10.9) | |  |
|  | **Valencia** | 38(2.4) | 43(2.3) | 20(2.3) | | 31(2.3) | |  |
|  | **Granada** | 52(3.3) | 56(2.9) | 28(3.3) | | 39(2.9) | |  |
|  | **Gerona** | 21(1.3) | 14(0.7) | 9(1.1) | | 15(1.1) | |  |
| **Family History of Pca** | **No** | 289(93.5) | 300(91.7) | 134(95.7) | | 198(90.8) | | 0.291 |
|  | **yes** | 20(6.5) | 27(8.3) | 6(4.3) | | 20(9.2) | |  |
| **Smoking** | **Non-smoker** | 76(24.4) | 98(30.0) | 38(27.1) | | 61(27.9) | | 0.478 |
|  | **Former/current smoker** | 235(75.6) | 229(70.0) | 102(72.9) | | 158(72.1) | |  |
| **Diabetes** | **No** | 262(80.4) | 284(77.6) | 115(75.7) | | 183(76.6) | | 0.601 |
|  | **yes** | 64(19.6) | 82(22.4) | 37(24.3) | | 56(23.4) | |  |
| **hypertension** | **No** | 167(51.1) | 189(51.8) | 75(49.3) | | 114(47.9) | | 0.801 |
|  | **yes** | 160(48.9) | 176(48.2) | 77(50.7) | | 124(52.1) | |  |
| **BMI (kg/m2) *** | **mean(sd)** | 27.71(0.21) | 27.58(0.20) | 27.53(0.31) | | 27.55(0.24) | | 0.948 |
| **waist circumference (cm)** | **mean(sd)** | 101.52(0.58) | 102.12(0.55) | 101.97(0.84) | | 101.70(0.68) | | 0.888 |
| **Physical Activity in Leisure Time (METs)** | **mean(sd)** | 173.50(14.34) | 187.61(13.99) | 191.07(21.38) | | 172.41(17.09) | | 0.810 |
| **kilocalories ingested daily** | **mean(sd)** | 2078.05(38.54) | 1985.10(36.87) | 1961.29(56.77) | | 1934.19(44.78) | | 0.076 |
| **Alcohol (g/day)** | **mean(sd)** | 2.46(0.07) | 2.45(0.07) | 2.42(0.10) | | 2.55(0.08) | | 0.735 |
| **Red Meat (g/day)** | **mean(sd)** | 33.96(1.55) | 33.82(1.48) | 35.74(2.28) | | 32.42(1.80) | | 0.725 |
| **Fruits (g/day)** | **mean(sd)** | 325.80(12.94) | 335.47(12.38) | 328.86(19.07) | | 312.36(15.04) | | 0.699 |
| **Vegetables (g/day)** | **mean(sd)** | 175.92(7.27) | 174.75(6.95) | 160.20(10.70) | | 160.00(8.44) | | 0.339 |
| **Dairy foods (g/day)**** | **mean(sd)** | 335.93(10.88) | 329.82(10.41) | 307.57(16.03) | | 327.99(12.64) | | 0.535 |
| **Dietary calcium (mg/d)** | **mean(sd)** | 944.82(20.86) | 914.75(19.96) | 886.82(30.73) | | 884.32(24.24) | | 0.217 |
| **Dietary vitamin D (mg/d)** | **mean(sd)** | 2.95(0.10) | 2.84(0.09) | 3.13(0.14) | | 2.93(0.11) | | 0.401 |
|  |  |  |  |  | |  |  | |
|  |  | **Genetic Score** | | | | |  | |
|  |  | **Q1** | **Q2** | **Q3** | **Q4** | | **p** | |
| **Risk factors for breast cancer** | **Category** | **N(%)** | **N(%)** | **N(%)** | **N(%)** | |  | |
| **Age (years)** | **mean(sd)** | 58.89(0.72) | 58.99(0.65) | 59.06(0.96) | 58.53(0.74) | | 0.963 | |
| **Ethnicity or race** | **White /caucasian** | 329(100) | 408(100) | 186(100) | 313(100) | |  | |
| **Education** | **Less than primary school** | 334(20.9) | 391(21.1) | 192(23.5) | | 290(22.5) | | 0.331 |
|  | **Primary school** | 623(39.0) | 676(36.4) | 290(35.5) | | 465(36.0) | |  |
|  | **Secondary school** | 393(24.6) | 486(26.2) | 187(22.9) | | 323(25.0) | |  |
|  | **University** | 247(15.5) | 304(16.4) | 149(18.2) | | 212(16.4) | |  |
| **Recruitment province** | **Madrid** | 340(21.3) | 388(20.9) | 146(17.8) | | 242(18.8) | | 0.026 |
|  | **Barcelona** | 428(26.8) | 514(27.7) | 219(26.8) | | 377(29.2) | |  |
|  | **Navarra** | 68(4.3) | 105(5.7) | 43(5.3) | | 71(5.5) | |  |
|  | **Guipuzcoa** | 99(6.2) | 126(6.8) | 66(8.1) | | 104(8.1) | |  |
|  | **Leon** | 240(15.0) | 262(14.1) | 119(14.5) | | 145(11.2) | |  |
|  | **Asturias** | 54(3.4) | 95(5.1) | 38(4.6) | | 74(5.7) | |  |
|  | **Huelva** | 12(0.8) | 23(1.2) | 14(1.7) | | 15(1.2) | |  |
|  | **Cantabria** | 200(12.5) | 193(10.4) | 97(11.9) | | 146(11.3) | |  |
|  | **Valencia** | 53(3.3) | 56(3.0) | 29(3.5) | | 38(2.9) | |  |
|  | **Granada** | 86(5.4) | 85(4.6) | 39(4.8) | | 65(5.0) | |  |
|  | **Gerona** | 17(1.1) | 10(0.5) | 8(1.0) | | 13(1.0) | |  |
| **Height (cm)** | **mean(sd)** | 159.49(0.35) | 160.01(0.32) | 159.48(0.47) | 159.65(0.36) | | 0.663 | |
| **BMI (kg/m^2^)** | **<20** | 17(5.2) | 38(9.3) | 8(4.3) | 20(6.4) | | 0.430 | |
|  | **20-24.9** | 149(45.3) | 184(45.1) | 84(45.2) | 141(45.0) | |  | |
|  | **25-29.9** | 100(30.4) | 117(28.7) | 60(32.3) | 102(32.6) | |  | |
|  | **≥30** | 63(19.1) | 69(16.9) | 34(18.3) | 50(16.0) | |  | |
| **Smoking** | **Never** | 195(59.3) | 238(58.5) | 116(62.4) | 181(58.2) | | 0.871 | |
|  | **Former** | 52(15.8) | 64(15.7) | 31(16.7) | 46(14.8) | |  | |
|  | **Current** | 82(24.9) | 105(25.8) | 39(21.0) | 84(27.0) | |  | |
| **Alcohol consumption (drinks/week)** | **mean(sd)** | 3.13(0.27) | 3.09(0.24) | 2.85(0.36) | 3.57(0.27) | | 0.376 | |
| **Family history of breast cancer** | **No first-degree relative affected** | 257(90.2) | 324(90.3) | 153(92.7) | 244(86.8) | | 0.228 | |
|  | **First-degree relative affected** | 28(9.8) | 35(9.7) | 12(7.3) | 37(13.2) | |  | |
| **Age at menarche** | **<12** | 59(17.9) | 66(16.2) | 40(21.5) | 54(17.3) | | 0.765 | |
|  | **12-13** | 149(45.3) | 199(48.8) | 80(43.0) | 148(47.3) | |  | |
|  | **≥14** | 121(36.8) | 143(35.0) | 66(35.5) | 111(35.5) | |  | |
| **Parity** | **0** | 59(17.9) | 76(18.6) | 34(18.3) | 61(19.6) | | 0.828 | |
|  | **1-2** | 182(55.3) | 239(58.6) | 107(57.5) | 167(53.5) | |  | |
|  | **>2** | 88(26.7) | 93(22.8) | 45(24.2) | 84(26.9) | |  | |
| **Age at first delivery** | **<20** | 6(1.8) | 15(3.7) | 5(2.7) | 9(2.9) | | 0.933 | |
|  | **20-24** | 84(25.8) | 94(23.0) | 45(24.2) | 76(24.4) | |  | |
|  | **25-29** | 102(31.3) | 136(33.3) | 59(31.7) | 101(32.4) | |  | |
|  | **30-34** | 56(17.2) | 64(15.7) | 31(16.7) | 51(16.3) | |  | |
|  | **35-39** | 18(5.5) | 16(3.9) | 12(6.5) | 10(3.2) | |  | |
|  | **≥40** | 60(18.4) | 83(20.3) | 34(18.3) | 65(20.8) | |  | |
| **Menopausal status** | **Premenopause** | 101(30.7) | 119(29.2) | 53(28.5) | 88(28.1) | | 0.901 | |
|  | **Postmenopause** | 228(69.3) | 289(70.8) | 133(71.5) | 225(71.9) | |  | |
| **Age at menopause** | **<50** | 102(31.0) | 129(31.6) | 60(32.3) | 87(27.8) | | 0.711 | |
|  | **50-54** | 74(22.5) | 107(26.2) | 43(23.1) | 79(25.2) | |  | |
|  | **≥55** | 18(5.5) | 25(6.1) | 12(6.5) | 27(8.6) | |  | |
|  | **Not Known (or not remember)** | 135(41.0) | 147(36.0) | 71(38.2) | 120(38.3) | |  | |
| **Hormone replacement therapy** | **Never** | 290(88.1) | 367(90.0) | 166(89.2) | 274(87.5) | | 0.088 | |
|  | **Ever** | 20(6.1) | 30(7.4) | 14(7.5) | 32(10.2) | |  | |
|  | **Not Known (or not remember)** | 19(5.8) | 11(2.7) | 6(3.2) | 7(2.2) | |  | |

***BMI (kg/m2): Body Mass Index one year before**

****Dairy foods include milk, yogurt and cheese**
