# Supplementary material for: Pigmentation phototype and prostate and breast cancer in a select Spanish population—A Mendelian randomization analysis in the MCC-Spain study
Source: PLoS One. 2018 Aug 14;13(8):e0201750. doi: 10.1371/journal.pone.0201750 (PMC6091948; doi:10.1371/journal.pone.0201750)
Supplement: S6 Table — (DOCX) [file pone.0201750.s006.docx]

**S6 Table. Results of MR-Egger regression**

|  |  | Prostate Cancer | | | | Breast Cancer | | | |
| --- | --- | --- | --- | --- | --- | --- | --- | --- | --- |
| Exposición | Category | α | p | β | p | α | p | β | p |
| Hair color | Light brown hair | 0.009 (-0.046, 0.064) | 0.73 | -0.094 (-0.289, 0.102) | 0.33 | 0.017 (-0.038, 0.073) | 0.52 | -0.024 (-0.221, 0.172) | 0.80 |
|  | Blonde hair | -0.017 (-0.063, 0.028) | 0.42 | 0.054 (-0.026, 0.134) | 0.17 | 0.018 (-0.033, 0.068) | 0.47 | -0.032 (-0.115, 0.052) | 0.43 |
| Skin color | Light brown skin | 0.023 (-0.012, 0.059) | 0.19 | -0.112 (-0.173, -0.051) | 0.001 | -0.017 (-0.077, 0.044) | 0.57 | 0.11 (-0.031, 0.251) | 0.12 |
|  | Fair skin | 0.040 (-0.001, 0.082) | 0.06 | -0.113 (-0.177, -0.050) | 0.002 | -0.035 (-0.085, 0.016) | 0.17 | 0.108 (0.036, 0.180) | 0.006 |
| Eye color | Light brown/green | -0.017 (-0.056, 0.023) | 0.38 | 0.229 (0.026, 0.432) | 0.03 | 0.008 (-0.052, 0.069) | 0.77 | 0.037 (-0.177, 0.251) | 0.72 |
|  | Blue/grey | -0.008 (-0.057, 0.040) | 0.72 | 0.006 (-0.067, 0.079) | 0.87 | 0.023 (-0.030, 0.077) | 0.36 | -0.037 (-0.121, 0.047) | 0.36 |
| Freckles | Yes | -0.001 (-0.051, 0.048) | 0.95 | -0.034 (-0.172, 0.103) | 0.60 | -0.020 (-0.068, 0.028) | 0.39 | 0.161 (0.040, 0.283) | 0.01 |
| Behavior of the skin in the sun | I rarely get burned and then I get tanned | 0.027 (-0.010, 0.063) | 0.14 | -0.161 (0.249, -0.073) | 0.001 | -0.025 (-0.081, 0.031) | 0.36 | 0.155 (0.013, 0.230) | 0.03 |
|  | I get burned and then I get tanned | 0.036 (-0.003, 0.74) | 0.07 | -0.151 (-0.231, -0.071) | 0.001 | -0.022 (-0.068, 0.024) | 0.32 | 0.140 (0.0458, 0.233) | 0.006 |
|  | I get burned and almost never I get tanned | 0.048 (0.002, 0.093) | 0.04 | -0.155 (-0.245, -0.065) | 0.002 | -0.037 (-0.080, 0.006) | 0.09 | 0.162 (0.079, 0.245) | 0.001 |
